# Proceedings of the Annual Meeting of the Medical Society of the State of Georgia, Held at Augusta, April 8th and 9th, 1857

**Published:** 1857-05

**Authors:** Eben Hillyer

**Affiliations:** Secretary


*Proceedings of the Annual Meeting of the Medical Society of the State of Georgia, held in Augusta, April 8th and 9th,* 1857.

The Society assembled at 10½ o'clock, in the Presbyterian Reading Room, and was called to order by the 2d Vice President, Dr. S. W. Burney, of Forsyth. The Recording Secretary being absent, Dr. Eben Hillyer, of Atlanta, was requested to act as Secretary *pro tem.*

The following members were present:

Richard D. Arnold, of Savannah; Jo's. A. Eve, of Augusta; Lewis D. Ford, of Augusta; M. H. Oliver, of Atlanta; R. Q. Dickinson, of Albany; W. L. Jones, of Augusta; J. G. Howard, of Savannah; L. A. Dugas, of Augusta; Henry F. Campbell, of Augusta; R. C. Beack, of Augusta; Eben Hillyer, of Atlanta, J. M. Turner, of Augusta; A Means, of Oxford; S. W. Burney, of Forsyth.

The proceedings of the last Annual Meeting held in Macon, April 9th, 1856, were read and approved.

On motion, the Rules were suspended, and the following gentlemen, on written application, were duly elected members of the Society:

Prof. Joseph P. Logan, of Atlanta; Prof. Jesse Boring, of LaGrange; Prof. John W. Jones, of Atlanta; Wm. S. Meiere, of Madison; John B. Hendrick, of Covington; G. L. McCleskey, of Madison; N. F. Powers, of Thompson; V. H. Taliaferro, of Atlanta; Olin S. Means, Oxford; T. C. H. Wilson, of Atlanta; E. J. Roach, of Pulaski; W. H. Doughty, of Augusta; W. L. Felder, of Augusta; W. T. Grant, of Columbia; R. H. Eaton, of Lawrenceville; M. J. Jones, of Warrington: A. T. Jenkins, of Green Co.; J. W. Gardner, of Augusta; J.

T. Dickinson, of Albany; S. S. Cranford, of Augusta; T. B. Ford, of Augusta; DeSaussure Ford, of Augusta; H. R. Casey, of Columbia; J. C. Carroll, of Lawrence Co.; E. B. Hook, of Augusta; E. H. W. Hunter, of Louisville; D. W. Marks, of Augusta; D. W. Young, of Augusta; H. H. Steiner, of Augusta.

The election of officers being next in order, a ballot was ordered, and the following gentlemen were duly elected for the ensuing year:

Dr. S. W. Burney, of Forsyth, President; Dr. H. F. Campbell, of Augusta, 1st Vice President; Dr. T. C. H. Wilson, of Atlanta, 2d Vice President.

On motion of Dr. Arnold of Savannah, the Corresponding and Recording Secretary, and Treasurer, were consolidated. A ballot was then ordered, and Dr. Eben Hillyer, of Atlanta, was declared unanimously elected.

The selection of Delegates to the American Medical Association being next in order, a committee of five, consisting of the following gentlemen—Drs. Dickinson, Dugas, Arnold, Taliaferro and Means,—were appointed by the President, to select them, and report at their earliest convenience.

Society then adjoured until three o'clock, P. M.

---

AFTERNOON SESSION.

Society called to order by the President. Reports from auxiliary societies being called for, Dr. E. Hillyer presented a report of the organization of officers and members of the auxiliary society in the City of Atlanta; which, upon motion, was received and adopted, and ordered to be put among the records of the Society.

The committee to appoint delegates to the American Medical Association, reported the names of the following gentlemen:

Dr. W. S. Meiere, of Madison; Dr. J. G. Howard, of Savannah; Dr. Jesse Boring, of La Grange; Dr. Joseph P. Logan, of Atlanta; Dr. Wm. S. Jones, of Augusta; Dr. Geo. F. Cooper, of Americus; Dr. N. F. Powers, of Thompson; Dr. Eben Hillyer, of Atlanta; Dr. T. S. Powell, of Sparta; Dr. R. D. Arnold, of Savannah; Dr. H. R. Casey, of Apling; Dr. Henry Gaither, of Oxford; Dr. S. W. Burney, of Forsyth.

The committee also reported the following:

*Resolved*, That should any of the gentlemen appointed be unable to attend, that they be authorized to appoint their own alternate.

The report was received and unanimously adopted.

The reading of Essays being next in order, Dr. Kollock, of

Savannah, read a very elaborate and interesting paper upon Vesico Vaginal Fistula.

Society then adjourned until 10 o'clock, Thursday morning.

---

THURSDAY MORNING, APRIL 9TH.

Society met pursuant to adjournment.

Minutes read and approved.

Upon written applications, the following gentlemen were elected members: Drs. W. T. Hollingsworth, of Morgan; J. W. Dent, S. W. Lamar, C. Walton, Charles Palmedo, of Augusta, and Thos. S. Powell, of Sparta.

On motion of Dr. Means, it was

*Resolved*, That those gentlemen of the Society who were appointed to read Essays at the present meeting, and may have prepared them, but who have been unavoidably prevented from attending, be requested to furnish their papers at their earliest convenience, to the Editor of the Southern Medical and Surgical Journal, for publication.

On motion, for the benefit of new members, the Secretary was required to read the Constitution and its amendments; also that the Editor of the Southern Medical and Surgical Journal be requested to publish the same in the columns of said Journal.

Dr. Jos. A. Eve, by appointment, read a paper upon the diseases of the Cervix Uteri, which was listened to with much interest by the Society.

Dr. L. A. Dugas contributed a full and interesting paper upon Fractures of the Scapula.

Dr. Dickinson, of Albany, offered the following, which was adopted:

*Resolved*, That the thanks of the Society be tendered Drs. Kollock, Eve and Dugas for the Essays read by them before the Society, and that they be requested to furnish copies for publication in the Southern Medical and Surgical Journal.

---

AFTERNOON SESSION.

Society called to order by the President. Dr. Hunter offered the following:

*Resolved*, That O. S. Proffett having been found to have been ineligible at the time of his election to membership in this Society, the Secretary is hereby instructed to erase his name from the list of members, which was unanimously adopted.

A Committee consisting of Drs. Means, Grant, Ford, Jones, and Campbell, were appointed to select subjects, and appoint Essayists for the next Annual Meeting.

The selection of the place for holding the next Annual

Meeting being now in order, a ballot was ordered, and upon counting the votes, it was found that Madison, Morgan County had received a majority. Dr. Thos. S. Powell, of Sparta, was elected Orator for the next annual meeting, and W. S. Meiere, of Madison, his alternate, should he be unable to attend. The Committee on Essays made the following report of subjects and Essayists for the next meeting, which was received and adopted:—Dr. J. G. Howard, of Savannah, on Uterine Diseases; Dr. E. J. Roach, of Pulaski, on the Propriety of Surgical Operations about the Joints; Dr. H. F. Campbell, of Augusta, on the Rectal Administration of Medicines; Dr. J. M. Green, on the Value of Escharotics in the Treatment of Cancer; Dr. R. D. Arnold, of Savannah, on the Pathology and Treatment of Yellow Fever; Dr. Ira E. Dupree, of Twiggs, on the Treatment of Prolapsus Uteri; Dr. Eben Hillyer, of Atlanta, on the Physiology of Menstruation; Dr. V. H. Taliaferro, of Atlanta, on Obstetrical Surgery; Dr. N. F. Powers, of Thompson, on Diseases of the Skin; Dr. Meiere, of Madison, on the Use of Alcohol in Typhoid Fever; Dr. R. Campbell, of Augusta, on Wounds of the Abdomen; Dr. J. P. Garvin, of Augusta, on Nervous Irritation of the Stomach. Voluntary Communications from any member of the Society are earnestly requested and will be gratefully received.

The following, by Dr. W. T. Grant, was adopted:

*Resolved*, 1st. That the Medical Society of the State of Georgia, return their sincere thanks to the Trustees of the Presbyterian Church, for their kindness in extending the convenience of their lecture room to the Society. 2d. That the thanks of the Society be extended to the profession and the citizens of Augusta for their liberal hospitality and kind reception of the members of the Society.

Dr. Campbell offered the following, which was adopted:

*Resolved*, That the funds of the Society now on hand, as by the report of the late Treasurer, be placed in the hands of the President and Treasurer, to be used in procuring such artistical illustrations as may be deemed necessary for the articles published under the auspices of the Society.

The following gentlemen were appointed on the Committee of Arrangements for the next meeting:

Drs. H. J. Oglesby, E. E. Jones, John B. Cranford, and G. L. McCleskey.

Dr. Hunter offered the following:

*Resolved*, That thanks of this Society be tendered the President and Secretary for the faithful manner in which they have discharged their respective duties.

The following by Dr. Arnold, which was adopted:

*Resolved*, That the thanks of the Society be tendered to the press of the city for the courtesy extended to it as a body.

A motion was passed instructing the Secretary to have published the resolutions of thanks to the Trustees of the Presbyterian Church, the Physicians, citizens, and press of Augusta, in the Augusta papers.

Dr. Grant offered the following:

*Resolved*, That this Society do now adjourn until the second Wednesday in April, 1858, to reassemble in the town of Madison, Morgan County, Georgia.

EBEN HILLYER, M. D., *Secretary.*

